# Inpatient Treatment for Uncomplicated and Complicated Acute Pelvic Inflammatory Disease: Ampicillin/Sulbactam Vs. Cefoxitin

**DOI:** 10.1155/S1064744993000286

**Published:** 1993

**Authors:** David L. Hemsell, George D. Wendel, Patricia G. Hemsell, Molly L. Heard, Brenda J. Nobles

**Affiliations:** 1 Department of Obstetrics and Gynecology University of Texas Southwestern Medical Center at Dallas and Parkland Memorial Hospital Dallas TX USA; 2 Department of Obstetrics and Gynecology University of Texas Sout-hwestern Medical Center at Dallas 5323 Harry Hines Boulevard, G6.226 Dallas TX 75235-9032 USA

## Abstract

*Objective:* Ampicillin plus sulbactam, an irreversible β-lactamase inhibitor, was compared to cefoxitin in the treatment of women with acute pelvic inflammatory disease (PID) with and without inflammatory mass(es).

*Methods:* Participation in an open, prospective, randomized clinical trial was offered to all women given the clinical diagnosis of acute PID who required inpatient therapy. *Neisseria gonorrhoeae* and *Chlamydia trachomatis* were sought in cervical and endometrial samples and aerobic and anaerobic species were sought in endometrial samples prior to treatment initiation. Treatment was given on at least 4 days and until women were afebrile for at least 48 h. Daily examinations were performed to assess response to therapy and safety. Only women in whom *C. trachomatis* was identified were discharged from the hospital on oral doxycycline to be taken for 10–14 days.

*Results:* One hundred twenty-four women were evaluated for safety; 117 (94%) were evaluated for efficacy. Demographic characteristics were similar for women in each treatment group. *N. gonorrhoeae* was recovered from 59% and *C. trachomatis* was recovered from 42% of study subjects. Inflammatory masses were identified in 35/76 (46%) women given ampicillin/sulbactam and 17/41 (41%) women given cefoxitin. Ampicillin/sulbactam cured 75 ,of 76 women (98.7%) [95% confidence interval (CI) 92.9–100.0%] and cefoxitin cured 37 of,41,omen (90.2%) (95% CI 76.9–97.3%) in that treatment regimen.

*Conclusions:* Overall ampicillin/sulbactam was more effective (*P* = 0.05) than cefoxitin, due to superior efficacy in infection complicated by inflammatory mass(es).35/35 vs. 12/17 cured; *P* =  0.007).


Infectious Diseases in Obstetrics and Gynecology 1:123–129 (1993)

© 1994 Wiley-Liss, Inc.

### Inpatient Treatment for Uncomplicated and Complicated Acute Pelvic Inflammatory Disease: Ampicillin/Sulbactam Vs. Cefoxitin

**David L. Hemsell, George D. Wendel, Jr., Patricia G. Hemsell, Molly L. Heard, and Brenda J. Nobles**

*Department of Obstetrics and Gynecology, University of Texas Southwestern Medical Center at Dallas, and Parkland Memorial Hospital, Dallas, TX*

### Abstract

***Objective:*** Ampicillin plus sulbactam, an irreversible β-lactamase inhibitor, was compared to cefoxitin in the treatment of women with acute pelvic inflammatory disease (PID) with and without inflammatory mass(es).

***Methods:*** Participation in an open, prospective, randomized clinical trial was offered to all women given the clinical diagnosis of acute PID who required inpatient therapy. *Neisseria gonorrhoeae* and *Chlamydia trachomatis* were sought in cervical and endometrial samples and aerobic and anaerobic species were sought in endometrial samples prior to treatment initiation. Treatment was given on at least 4 days and until women were afebrile for at least 48 h. Daily examinations were performed to assess response to therapy and safety. Only women in whom *C. trachomatis* was identified were discharged from the hospital on oral doxycycline to be taken for 10–14 days.

***Results:*** One hundred twenty-four women were evaluated for safety; 117 (94%) were evaluated for efficacy. Demographic characteristics were similar for women in each treatment group. *N. gonorrhoeae* was recovered from 59% and *C. trachomatis* was recovered from 42% of study subjects. Inflammatory masses were identified in 35/76 (46%) women given ampicillin/sulbactam and 17/41 (41%) women given cefoxitin. Ampicillin/sulbactam cured 75 of 76 women (98.7%) [95% confidence interval (CI) 92.9–100.0%] and cefoxitin cured 37 of 41 women (90.2%) (95% CI 76.9–97.3%) in that treatment regimen.

***Conclusions:*** Overall ampicillin/sulbactam was more effective ($P = 0.05$) than cefoxitin, due to superior efficacy in infection complicated by inflammatory mass(es) 35/35 vs. 12/17 cured; $P = 0.007$). © 1994 Wiley-Liss, Inc.

Key words
Pelvic inflammatory disease, inflammatory mass(es), antibiotic therapy

A variety of broad-spectrum single-agent and combination regimens have been highly successful when given to women hospitalized for treatment of acute pelvic inflammatory disease (PID). The optimal antibiotic regimen for inpatient therapy of such women has not been established. The etiology of this acute pelvic infection is generally thought to involve *Neisseria gonorrhoeae*, *Chlamydia trachomatis*, and a wide variety of anaerobic and aerobic bacteria. Treatment regimens must include broad-spectrum coverage of these likely pathogens. Additionally, therapy must be effective in the various forms and complications of PID (e.g., endometritis, salpingitis, and pelvic inflammatory mass/abscess). Combination antimicrobial therapy has been recommended by the Centers for Disease Con-

Address correspondence/reprint requests to Dr. David L. Hemsell, Department of Obstetrics and Gynecology, University of Texas Southwestern Medical Center at Dallas, 5323 Harry Hines Boulevard, G6.226, Dallas, TX 75235-9032.

Received August 23, 1993

Accepted December 2, 1993

trol and Prevention (CDC) since 1982.[1] However, many single-agent therapeutic regimens, including cefoxitin, have been successful in the treatment of women with PID.[2,3] Ampicillin alone was shown to be effective in one small clinical trial.[4] Since many potentially significant pathogens of pelvic infection produce β-lactamase enzyme, the addition of a suicide-type β-lactamase inhibitor to ampicillin should enchance and extend its antibacterial activity.

A prospective, randomized, open clinical trial was designed in which women with clinical symptoms and signs of acute PID who required hospitalization for therapy would be treated with intravenous (IV) ampicillin/sulbactam or cefoxitin. This report details results of early (inpatient) therapy only.

### SUBJECTS AND METHODS

Women 18 years of age or older who presented to the emergency room of Parkland Memorial Hospital complaining of lower abdominal and/or pelvic pain and who had abdominal, cervical motion, uterine, and adnexal tenderness were considered for inclusion in this clinical trial. They also had to have one or more of the following: temperature >38°C; leukocyte count $>10{,}000/mm^3$; Gram-negative diplococci in the endocervix; pelvic inflammatory mass(es); or leukocytes and bacteria from a peritoneal specimen. Pregnant or breastfeeding women were excluded as were those who were suspected to have a ruptured abscess; required concomitant antimicrobial therapy; had a history of known hypersensitivity reaction to cephalosporins, penicillin, or tetracyclines; had received successful antibiotic therapy within the 4 days preceding consideration for study entry; were receiving another investigational drug; had impaired immunological function and/or leukopenia ($<1{,}500/mm^3$); and had clinically significant renal dysfunction. Women who met the inclusion and exclusion criteria and who signed an Institutional Review Board-approved consent form were sequentially enrolled in this clinical trial.

Prior to antimicrobial administration, material recovered from the endocervix with a sterile swab was cultured for *N. gonorrhoeae* and tested for *C. trachomatis* by culture and/or for fluorescent antibody detection by MicroTrak® (Syva Company, San Jose, CA). An endometrial sample also was obtained using a Pipelle® (Unimar, Wilton, CT) for identification of *N. gonorrhoeae, C. trachomatis,* and aerobic and anaerobic bacteria in the departmental research microbiology laboratory. *N. gonorrhoeae* specimens were plated directly onto Martin-Lewis Transgrow® media (Remel, Lenexa, KS) for identification using the quadFERM® system (Analytab Products, Plainview, NY). *C. trachomatis* specimens were placed into refrigerated Multi-Microbe® medium (MicroTest, Inc., Snellville, GA) for transport and cultured in McCoy® cells (Ortho Diagnostic, Ranton, NJ). Identification was made using the Syva® confirmation test (Syva Company). Gram-negative aerobic isolates were identified using the API 20-E® system (Analytab Products) and conventional methods were employed for identification of Gram-positive aerobic bacteria. Anaerobic isolates were processed in a Coy® anaerobic glove box (Coy Laboratory Products, Ann Arbor, MI) on standard media and identified using the API 20-A® system (Analytab Products). Agar dilution susceptibility testing by minimal inhibitory concentration was performed using National Committee for Clinical Laboratory Standards (NCCLS) guidelines for aerobic (M7-T2) and anaerobic (M11-A) bacteria. Standard reference powder of each antibiotic was provided by the manufacturer. β-Lactamase was detected using a nitrocephin disk (Becton-Dickinson, Cockeyville, MD).

The following clinical laboratory studies were performed prior to the initiation of therapy, at the completion of successful therapy, when therapy failed, or when patients left the hospital against medical advice (AMA): complete blood count (CBC) with differential; hepatic and renal function tests; direct Coombs test; prothrombin time (PT); partial thromboplastin time (PTT); and urinalysis.

Women treated under this protocol were given consecutive study numbers as they were enrolled, and treatment was randomly assigned according to a 2:1 (ampicillin/sulbactam:cefoxitin) computer-generated randomization schedule. The regimen doses for ampicillin/sulbactam and cefoxitin were 3 and 2 g, respectively. Each therapeutic regimen was to be administered IV every 6 h on at least 4 consecutive days and until the patient was afebrile for at least 48 h. Sonography was performed on all study subjects to identify and document inflammatory pelvic mass(es). Results were interpreted by 1 of 4 faculty sonographers using the criteria of a complex enlargement that was predominantly sonolucent and septated with irregular borders in the region of an adnexa without an ovary detected separately. Doppler flow imaging was not performed. Women with *C. trachomatis* at study entry were given 100-mg doxycycline tablets to be taken orally twice daily for 10–14 days when discharged from the hospital. Subjects negative for *C. trachomatis* were discharged on no oral antibiotic.

The study subjects were evaluated clinically twice daily during hospitalization. Clinical cure was defined as resolution of abnormal tenderness, disappearance of temperature elevation for at least 48 h, normalization of the leukocyte count, and no requirement for other antimicrobial or surgical therapy for the original infection. It was necessary to receive a regimen for at least 48 h to be evaluable for efficacy. Clinical failure was defined as persistent or increasing symptoms, physical findings, and temperature after at least 48 h of the original regimen necessitating alternative antimicrobial or surgical therapy.

A power analysis indicated that 120 study subjects (2:1:ampicillin/sulbactam: cefoxitin) would be required to have an 80% chance of detecting a difference in efficacy of 20%, assuming an efficacy of 95% for ampicillin with sulbactam. Data were analyzed utilizing two-tailed Fisher's exact, log likelihood $\chi^2$, Welch's approximation, and Student's t-tests. Exact confidence intervals (CI) were calculated. Clinical significance was defined as $P \leqslant 0.05$.

### RESULTS

One hundred twenty-four women were entered into this clinical trial over 37 months; 117 (94%) were evaluable for clinical efficacy determination. Seven women, 1 given cefoxitin and 6 given ampicillin/sulbactam, were exluded from clinical evaluation. The woman given cefoxitin was treated for subacute bacterial endocarditis. Three women given ampicillin/sulbactam left the hospital AMA and 2 others were given additional antimicrobial treatment for syphilis and *Trichomonas vaginalis* vaginitis prior to completion of protocol therapy. One other study subject experienced recurrent pruritus which began on her third day of ampicillin/sulbactam therapy; she was clinically improving and was treated for 2 more days with clindamycin and gentamicin. Study numbers for these subjects were 5, 41, 44, 65, 86, 112, and 123.

**TABLE 1. Demographic and contraception characteristics of 117 hospitalized women with acute PID[a]**

| Characteristic | Treatment regimen: Ampicillin/sulbactam (N = 76) | Cefoxitin (N = 41) |
|---|---|---|
| Age (years) | 24.0 ± 4.7 | 24.5 ± 5.9 |
| Height (in.) | 64.0 ± 2.7 | 64.0 ± 3.2 |
| Weight (lb) | 139.1 ± 41.9 | 142.7 ± 30.1 |
| Gravidity | 1.2 ± 1.4 | 1.1 ± 1.8 |
| Parity | 0.8 ± 1.1 | 0.9 ± 1.8 |
| Race | | |
| Black | 58 (76) | 31 (76) |
| Caucasian | 10 (13) | 7 (17) |
| Hispanic | 8 (11) | 3 (7) |
| Contraception | | |
| None | 51 (67) | 32 (78) |
| Oral contraceptives | 11 (14) | 6 (15) |
| Barrier | 7 (9) | 1 (2) |
| Tubal ligation | 5 (7) | 1 (2) |
| Intrauterine device | 2 (3) | 1 (2) |
| Previous PID | 14 (18) | 9 (22) |
| Previous *N. gonorrhoeae* | 25 (33) | 10 (24) |

[a] Data are presented as mean ± standard deviation or number with percent in parentheses.

Demographic, contraceptive, and prior infection variables for the evaluable women were statistically similar and are presented by therapeutic regimen in Table 1. Infection-related variables (Table 2) were also similar, except for a higher mean temperature reading at study entry in women given ampicillin/sulbactam. Of the 52 women with inflammatory mass(es), 24 (46%) denied a history of PID or positive culture for *N. gonorrhoeae*.

Excluding *N. gonorrhoeae* and *C. trachomatis*, 489 aerobic and anaerobic bacteria were recovered from endometrial specimens obtained from these 117 evaluable women (Table 3). The predominant aerobic endometrial isolates were Gram-positive organisms (86%); peptostreptococci accounted for 44% of anaerobic isolates. *Prevotella bivius* was the most frequently isolated Gram-negative anaerobe, accounting for 23% of all identified anaerobic isolates. The median number of isolates per patient was 5 organisms. One hundred fifteen isolates (23%) produced β-lactamase. β-Lactamase production was observed most frequently in enterobacteri-

TABLE 2. Entry infection-related variables for 117 women treated for acute PID[a]

| Clinical variable | Treatment regimen: Ampicillin/sulbactam (N = 76) | Cefoxitin (N = 41) |
|---|---|---|
| Duration of pain (days) | 3.9 ± 4.7 | 4.0 ± 4.1 |
| Interval to last menstrual period (days) | 12.5 ± 10.1 | 16.1 ± 18.7 |
| Leukocyte count (× $10^3/mm^3$) | 16.4 ± 6.0 | 17.1 ± 5.4 |
| Hemoglobin (g %) | 12.6 ± 1.6 | 12.7 ± 1.5 |
| Temperature (°C)* | 38.5 ± 0.7 | 38.2 ± 0.8 |
| Inflammatory mass(es) | 35 (46) | 17 (41) |
| <6 cm size | 15 (43) | 8 (47) |
| 6–10 cm size | 17 (49) | 7 (41) |
| ≥10 cm size | 3 (8) | 2 (12) |

[a] Data are presented as mean ± standard deviation or number with percent in parentheses.
*$P = 0.04$.

TABLE 3. Endometrial isolates recovered prior to therapy of women with acute PID[a]

| Bacterial species/group | No. of isolates | β-Lactamase positive isolates | MIC 90[b]: Ampicillin/sulbactam | Cefoxitin |
|---|---|---|---|---|
| *Neisseria gonorrhoeae*[c] | 69 | 3 (4) | 1 | 1 |
| *Chlamydia trachomatis*[c] | 49 | | | |
| *Staphylococcus epidermidis* | 78 | 13 (17) | 1 | 2 |
| *Staphylococcus aureus* | 10 | 3 (30) | 1 | 4 |
| *Streptococcus* species | 46 | | ≤0.25 | 2 |
| *Streptococcus agalactiae* | 27 | | ≤0.25 | 2 |
| *Enterococcus faecalis* | 15 | | 1 | ≥128 |
| *Escherichia coli* | 21 | 9 (43) | 32 | 4 |
| *Proteus* species | 2 | | 0.5 | 2 |
| *Pseudomonas aeruginosa* | 2 | | ≤0.25 | 1 |
| *Klebsiella pneumoniae* | 1 | 1 (100) | 4 | 2 |
| *Citrobacter diversus* | 1 | | ≤0.25 | 0.5 |
| *Acinetobacter calcoaceticus* var. *anitratus* | 1 | 1 (100) | 0.5 | 64 |
| *Peptostreptococcus* species | 126 | 1 (1) | 1 | 4 |
| *Clostridium* species | 4 | | 0.5 | 4 |
| *Propionibacterium* species | 11 | 1 (9) | 1 | 8 |
| *Prevotella/Bacteroides* species | 66 | 34 (52) | 1 | 4 |
| *Prevotella bivius* | 51 | 33 (65) | 2 | 2 |
| *Bacteroides fragilis* | | | | |
| *fragilis* | 6 | 6 (100) | 4 | 8 |
| *distasonis* | 1 | | 16 | 32 |
| *thetaiotaomicron* | 2 | 2 (100) | 1 | 32 |
| *vulgatus* | 3 | 3 (100) | 4 | 8 |
| *Prevotella disiens* | 7 | 3 (43) | ≤0.25 | 0.5 |
| *Fusobacterium* species | 8 | 2 (25) | 0.5 | 1 |

[a] Data are presented as number or number with percent in parentheses.
[b] Antibiotic concentration in μg/ml inhibiting 90% of bacterial species.
[c] Patients with recovery at endocervical and/or endometrial sites.

aceae and *Prevotella* or *Bacteroides* species. Only 3 gonococcal strains (4%) produced β-lactamase.

The recovery of *N. gonorrhoeae* and *C. Trachomatis* from the endocervix and endometrium was similar in both treatment regimens (data not shown). Of the 117 evaluable women, 40 (34%) had *N. gonorrhoeae* only, 20 (17%) had *C. trachomatis* only, 29 women (25%) had both microorganisms, and 28 women (24%) had neither of these species. Of the 69 women (59%) with *N. gonor-*

TABLE 4. Clinical outcome of 117 women treated for acute PID[a]

| Outcome | Treatment regimen | |
|---|---|---|
| | Ampicillin/sulbactam | Cefoxitin |
| Success without mass | 40/41 (97.6) | 24/24 (100) |
| Days of therapy | 4.4 ± 0.9 | 4.3 ± 0.6 |
| Success with mass* | 35/35 (100) | 13/17 (76.5) |
| Days of therapy** | 4.9 ± 1.1 | 6.0 ± 2.1 |
| Overall success** | 75/76 (98.7) | 37/41 (90.2) |
| 95% CI | 92.9–100.0 | 76.9–97.3 |
| Days of therapy | 4.6 ± 1.0 | 5.0 ± 1.6 |

[a]Data are presented as mean ± standard deviation or number of patients cured/number of patients treated with percent in parentheses.
*$P = 0.007$.
**$P = 0.05$.

*rhoeae*, 61 (88%) had isolates recovered from both cervical and endometrial sites, 6 (9%) had isolates only in the cervix, and 2 (3%) had isolates recovered only from endometrial specimens. The same recovery rates by site for the 49 women (42%) with *C. trachomatis* were 33 (68%), 9 (18%), and 7 (14%), respectively. *N. gonorrhoeae* was found in both the cervix and endometrium of infected women more frequently ($P = 0.015$) than was *C. trachomatis*. The absence of *N. gonorrhoeae* and/or *C. trachomatis* was associated with failure of the original regimen ($P = 0.011$).

Clinical outcome data for the study subjects are presented in Table 4. The overall rate of cure was higher for women treated with ampicillin/sulbactam, due primarily to its efficacy in the treatment of PID complicated by inflammatory mass(es). All 5 women who failed initial therapy required alteration in their therapy, which they received for a mean duration of 9 days. All 4 of the cefoxitin failures occurred in women with inflammatory masses of 6 cm or larger, and 1 of the 4 women required abscess drainage by colpotomy. Only 1 of the 5 treatment failures had either *N. gonorrhoeae* or *C. trachomatis*; she had both pathogens, an inflammatory mass larger than 6 cm, and was in the cefoxitin group. The woman who ultimately failed therapy with ampicillin/sulbactam had therapy altered on day 6 of therapy. Her temperature was 38.3°C, and she had moderate tenderness. Her therapy was altered to clindamycin and gentamicin, and she was discharged 3 days later. Her admission leukocyte count was $38.3 \times 10^3/mm^3$, and it was $20.5 \times 10^3/mm^3$ at therapy alteration. Cefoxitin failures were diagnosed after a mean duration of therapy of 5 days (range 4–7 days) with a mean temperature of 38.3°C (range 38.1–38.5°C) at therapy alteration. The altered therapy (clindamycin, gentamicin, and ampicillin) was administered for a mean of 4 days (range 3–6 days) before discharge from the hospital.

Clinical and laboratory adverse events were not clinically serious and were comparable for women in each antibiotic treatment regimen (Table 5). Only the previously mentioned women with an allergic reaction to ampicillin/sulbactam had an adverse reaction dictating removal from the study protocol.

### DISCUSSION

According to data from the CDC through 1991,[5] the reported number of women from whom *N. gonorrhoeae* was recovered has been steadily decreasing over the past 10 years in the United States. The number of women hospitalized for therapy of acute PID has decreased even more markedly over the same time period.[6] The explanation for these trends in the United States is unclear, especially since rates of syphilis have increased during the same period.[7] *N. gonorrhoeae* continues to be the sexually transmitted disease species most frequently recovered from women admitted to our hospital for parenteral therapy. Although the percentage of women admitted for inpatient therapy who had *N. gonorrhoeae* has remained relatively constant at between 50 and 60% over the past 7 years (data not shown), the number of women admitted has decreased almost 70% from 226 in 1985 to only 60 in 1992.

We found that the broad-spectrum antimicrobial regimen of ampicillin/sulbactam given 3g IV every 6 h was clinically superior to cefoxitin 2 g IV every 6 h in the treatment of women with acute PID diagnosed in an emergency room. On subgroup analyses, the efficacy of the 2 regimens was similar for uncomplicated PID. However, in women with infection complicated by pelvic inflammatory mass(es) detected by physical and ultrasound examinations, ampicillin/sulbactam had significantly greater efficacy. Furthermore, in this latter group the mean number of days of therapy for women treated with ampicillin/sulbactam was less than that of women treated with cefoxitin, suggesting a more rapid therapeutic response.

Not unexpectedly, the treatment failures were

TABLE 5. Adverse events in 124 women treated as inpatients for acute PID[a]

| Adverse event | Treatment regimen Ampicillin/Sulbactam (N = 82) | Cefoxitin (N = 42) |
|---|---|---|
| Clinical | | |
| Diarrhea | 3 (4) | 4 (10) |
| Pruritus | 2 (2) | 2 (5) |
| IV site pain/edema | 2 (2) | 0 (0) |
| IV site phlebitis | 1 (1) | 1 (2) |
| Rash | 0 (0) | 1 (2) |
| Laboratory test[b] | | |
| Aspartate aminotransferase | 3 (4) | 3 (7) |
| Alkaline phosphatase | 3 (4) | 1 (2) |
| PTT | 1 (1) | 1 (2) |

[a] Data are presented as number with percent in parentheses.
[b] Elevation out of normal range at end of therapy with normal value prior to study entry.

generally associated with non-gonococcal, non-chlamydial infection and large (≥6 cm) pelvic inflammatory masses at enrollment. Almost half of these women denied previous pelvic infection, indicating that inflammatory mass formation can complicate an initial infection. All of the cefoxitin-treated failures were women with pelvic masses. In this protocol, we followed our usual practice of clinically following all women after treatment for PID, especially to detect resolution or persistence of inflammatory mass(es). In a previous clinical trial,[10] only 12% of women had a persistent adnexal mass that required surgical diagnosis/therapy. A sterile hydrosalpinx was found in each instance.

Neither of the regimens presented here is recommended for adequate treatment of *C. trachomatis* infection. Previous investigators have confirmed that β-lactam antimicrobial agents have clinical efficacy in acute PID, but may fail to eradicate *C. trachomatis*.[8,9] Clinical success of initial regimens not active against chlamydia has been observed in patients treated in our hospital under prospective protocols.[10–13] If the addition of doxycycline to PID regimens is only to eradicate *C. trachomatis*, it seems appropriate to identify those women and provide specific therapy. If testing is not done, all women should be treated. Administration of doxycycline for acute PID did not prevent adhesion formation or tubal occlusion in a report by Teisala and coworkers.[14] In that report, women with *C. trachomatis* prior to treatment with penicillin and metronidazole did not have it when retested at second-look laparoscopy. According to Westrom and coinvestigators,[15] tetracycline treatment did not result in a significantly lower incidence of post-infection tubal occlusion in an earlier report on a large number of Scandinavian women treated for PID. All women in both treatment regimens were given oral doxycycline at hospital discharge if they had chlamydia at enrollment into the clinical trial. Experimental polymerase chain reaction (PCR)-based testing appears to be more sensitive and specific than currently available techniques, including culture. When developed and available, it may provide the most exact means of identifying *C. trachomatis* in clinical settings.

There are several limitations to this study. All diagnoses were clinical and not laparoscopic. In 1969 Jacobson and Westrom[16] reported that an accurate clinical diagnosis of acute PID was made in only 65% of women undergoing laparoscopy. Hadgu and coworkers[17] reported that, if women were young, unmarried, had vaginal discharge, fever, tender adnexal swelling, an elevated erythrocyte sedimentation rate, and *N. gonorrhoeae* in the cervix, the probability of the accuracy of the clinical diagnosis was 97%. Cumming et al.[18] suggested that normal laparoscopic findings did not exclude significant endosalpingeal infection, and Sellors and colleagues[19] reported that laparoscopic visualization of the fallopian tube was 50% sensitive with 80% specificity for the diagnosis of salpingitis when fimbrial histopathology was utilized to establish the diagnosis. In our opinion, data do not support routinely performing laparoscopy in all women given the clinical diagnosis of PID using criteria proposed by Hager and coworkers,[20] which were utilized in this clinical trial.

Follow-up endocervical and endometrial cultures were only performed for clinical treatment failures. Thus, the relation between bacteriologic and clinical cure was not examined. This report does not address long-term clinical response and fertility, both important components in long-term efficacy analyses. The potential for biased selection for entry did exist since this was an open clinical trial. All women given the diagnosis of acute PID who required inpatient therapy and met inclusion/exclusion criteria were approached for participation and consent. The 2 study groups were similar in terms of demography, clinical presentation, microbiologic spectrum of pelvic pathogens, and the frequency and relative sizes of inflammatory mass(es) were similar in both sets of patients. Thus, there appeared to be no selection biases in the hospitalized treatment groups.

In summary, we conclude that ampicillin/sulbactam was significantly more effective than cefoxitin for the inpatient treatment of acute PID, especially when complicated by pelvic inflammatory mass(es).

### ACKNOWLEDGMENTS

This work was supported in part by a Grant-in-Aid from Roerig Pharmaceuticals.

### REFERENCES

1. Centers for Disease Control: Sexually transmitted diseases treatment guidelines 1982. Morbidity and Mortality Weekly Report 31(Suppl 2):33S–60S, 1982.
2. Peterson HB, Walker CK, Kahn JG, Washington AE, Eschenbach DA, Faro S: Pelvic inflammatory disease. Key treatment issues and options. JAMA 266:2605–2611, 1991.
3. Peterson HB, Galaid EI, Zenilman JM: Pelvic inflammatory disease: Review of treatment options. Rev Infect Dis 12(Suppl 6): S656–S664, 1990.
4. Spence MR, Genadry R, Raffel L: Randomized prospective comparison of ampicillin and doxycycline in the treatment of acute pelvic inflammatory disease in hospitalized patients. Sex Transm Dis 8(Suppl 2):164–166, 1981.
5. Centers for Disease Control: Summary of notifiable diseases, United States, 1991. Morbidity and Mortality Weekly Report 40:24, 1991.
6. Centers for Disease Control: Pelvic inflammatory disease: Guidelines for prevention and management. Morbidity and Mortality Weekly Report 40:4, 1991.
7. Gersham KA, Rolfs RT: Diverging gonorrhea and syphilis trends in the 1980's: Are they real? Am J Public Health 81:1263–1266, 1991.
8. Sweet RL, Schachter J, Robbie MO: Failure of β-lactam antibiotics to eradicate *Chlamydia trachomatis* in the endometrium despite apparent clinical cure of acute salpingitis. JAMA 250:2641–2645, 1983.
9. Kousseim M, Ronald A, Plummer FA, D'Costa L, Brunham RC: Treatment of acute pelvic inflammatory disease in the ambulatory setting: Trial of cefoxitin and doxycycline versus ampicillin-sulbactam. Antimicrob Agents Chemother 35:1651–1656, 1991.
10. Hemsell DL, Santos-Ramos R, Cunningham FG, Nobles BJ, Hemsell PG: Cefotaxime treatment for women with community acquired pelvic abscesses. Am J Obstet Gynecol 151:771–777, 1985.
11. Hemsell DL, Hemsell PG, Heard ML, Nobles BJ: Piperacillin and a combination of clindamycin and gentamicin for hospital and community acquired acute pelvic infections including pelvic abscess. Surg Gynecol Obstet 165:223–229, 1987.
12. Hemsell DL, Nobles BJ, Heard MC, Hemsell PG: Upper and lower reproductive tract bacteria in 126 women with acute pelvic inflammatory disease: Microbial susceptibility and clinical response to four therapeutic regimens. J Reprod Med 33:799–805, 1988.
13. Hemsell DL, Wendel GD, Gall SA, Newton ER, Gibbs RS, Knuppel RA, Lane TW, Sweet RL: Multicenter comparison of cefotetan and cefoxitin in the treatment of acute obstetric and gynecologic infections. Am J Obstet Gynecol 158:722–728, 1988.
14. Teisala K, Heinonen PK, Aine R, Punnonen R, Paavonen S: Second look laparoscopy after treatment of acute pelvic inflammatory disease. Obstet Gynecol 69:343–346, 1987.
15. Westrom L, Iosif S, Svensson L, Märdh P-A: Infertility after acute salpingitis: Results of treatment with different antibiotics. Curr Ther Res 26:752–759, 1979.
16. Jacobson L, Westrom L: Objectivized diagnosis of acute pelvic inflammatory disease. Diagnostic and prognostic values of routine laparoscopy. Am J Obstet Gynecol 105: 1088–1098, 1969.
17. Hadgu A, Westrom L, Brooks CA, Reynolds GH, Thompson SE: Predicting acute pelvic inflammatory disease: A multivariate analysis. Am J Obstet Gynecol 155: 954–960, 1986.
18. Cumming DC, Honoré LH, Scott JZ, Williams KE: Microscopic evidence of silent inflammation in grossly normal fallopian tubes with ectopic pregnancy. Int J Fertil 33:324–328, 1988.
19. Sellors J, Mahony J, Goldsmith C, et al.: The accuracy of clinical findings and laparoscopy in pelvic inflammatory disease. Am J Obstet Gynecol 164:113–120, 1991.
20. Hager WD, Eschenbach DA, Spence MR, Sweet RL: Criteria for diagnosis and grading of salpingitis. Obstet Gynecol 61:113–114, 1983.
